# Reconstructing aerosol optical depth using spatiotemporal Long Short-Term Memory convolutional autoencoder

**DOI:** 10.1038/s41597-023-02696-w

**Published:** 2023-11-30

**Authors:** Lu Liang, Jacob Daniels, Michael Biancardi, Yuye Zhou

**Affiliations:** 1 Department of Geography and the Environment, University of North Texas, Denton, TX 76203 USA; 2 Department of Electrical Engineering, University of North Texas, Denton, TX 76203 USA; 3 Department of Computer Science and Engineering, University of North Texas, Denton, TX 76203 USA; 4 School of Architecture and Urban Planning, Nanjing University, Nanjing, 210093 China

**Keywords:** Atmospheric dynamics, Environmental impact, Atmospheric dynamics

## Abstract

Aerosol Optical Depth (AOD) is a crucial atmospheric parameter in comprehending climate change, air quality, and its impacts on human health. Satellites offer exceptional spatiotemporal AOD data continuity. However, data quality is influenced by various atmospheric, landscape, and instrumental factors, resulting in data gaps. This study presents a new solution to this challenge by providing a long-term, gapless satellite-derived AOD dataset for Texas from 2010 to 2022, utilizing Moderate Resolution Imaging Spectroradiometer (MODIS) Multi-angle Implementation of Atmospheric Correction (MAIAC) products. Missing AOD data were reconstructed using a spatiotemporal Long Short-Term Memory (LSTM) convolutional autoencoder. Evaluation against an independent test dataset demonstrated the model’s effectiveness, with an average Root Mean Square Error (RMSE) of 0.017 and an R^2^ value of 0.941. Validation against the ground-based AERONET dataset indicated satisfactory agreement, with RMSE values ranging from 0.052 to 0.067. The reconstructed AOD data are available at daily, monthly, quarterly, and yearly scales, providing a valuable resource to advance understanding of the Earth’s atmosphere and support decision-making concerning air quality and public health.

## Background & Summary

The measurement of Aerosol Optical Depth (AOD) is crucial for evaluating atmospheric aerosol loading. High-quality, long-term AOD data with temporal and spatial continuity is a critical parameter in several applications including air quality monitoring^1–3^, climate change^4–6^, public health^7,8^, and urban planning^9,10^. While satellite observations are an excellent source of data for quantifying aerosol dynamics in terms of spatiotemporal coverage^11^, they may be subject to various factors that can affect measurements such as cloud cover, surface brightness, instrument errors, and satellite orbit limitations resulting in data gaps^12–14^.

Creating a gapless, satellite-derived AOD dataset is highly desired by various stakeholders. Many techniques have been developed to fill in missing AOD data including data assimilation^11,15^, spatial or temporal interpolation^14,16,17^, and machine learning-based methods^18–21^. However, restoring data gaps in satellite-based AOD retrievals remains challenging due to thick cloud obstructions causing a large swath of missing information. This results in insufficient spatially neighboring data from which to interpolate or extrapolate to the missing regions. Additionally, the single-band nature of AOD data limits the usage of cross-band correlation. Furthermore, AOD fluctuates rapidly on a daily or even hourly time scale compared to more stable satellite imagery, making it difficult to rely on temporally-focused solutions^22^.

The use of state-of-the-art deep learning (DL) for quantitative AOD retrieval has emerged but is currently limited in the literature, largely due to the challenges of acquiring sufficient training samples^23^. Ground observation networks, such as the Surface Radiation Budget (SURFRAD) and AErosol RObotic NETwork (AERONET), provide excellent data sources of globally distributed spectral AOD observations^24,25^. Nevertheless, their limited spatial coverage makes them more suitable as test datasets rather than for training and validation purposes when developing fine spatial resolution DL models. For example, Texas only has 11 AERONET sites with sporadic data, and many locations lack data spanning over a year or two making this sample size insufficient to train a DL model to run data over a decade-long period. Although some studies have used computer programs to simulate artificial atmospheric artifacts^4,17^, these do not reflect reality, particularly when atmospheric interference tends to cluster or follow irregular patterns. To overcome these limitations, this study presents an effective method based on convolutional neural networks to reconstruct MODIS AOD data for an environmentally and socially significant region.

## Methods

### Study area

**Fig. 1:**
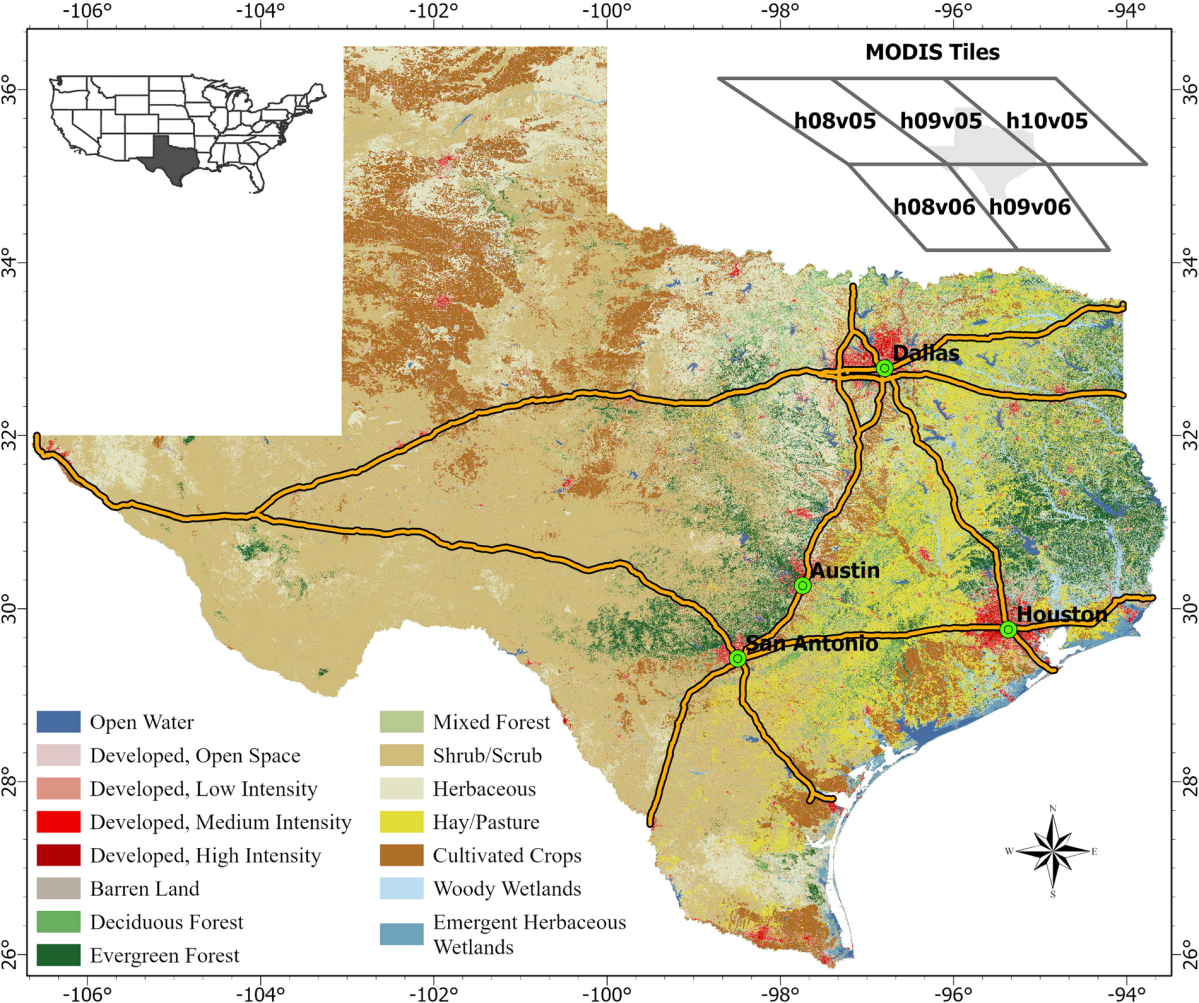
The heterogeneous land cover and land use types in the state of Texas. The top right inset shows the MODIS tile footprint covering Texas.

We selected the state of Texas as our study area for reconstructing AOD data as it spans 266,807 square miles and ranks as the second-largest state in the U.S. by both area and population. Texas boasts a diverse climate and landscape comprised of ten ecoregions, ranging from bottomland hardwood forests in the east to prairies and post oak savannah in the central region and high plains in the west. Geology, climate, and human activities shape the land cover diversity of Texas, where annual rainfall varies from eight inches in the deserts of far west Texas to 56 inches in the swamps of east Texas. According to the US Census Bureau, Texas’s population grew by more than 12 million between 1990 and 2020, representing an increase of over 70%^26^. The major highways crossing three major metropolitan areas of Houston, Dallas-Fort Worth, and San Antonio-Austin define the Texas Urban Triangle region (Fig. 1), which accounts for nearly 75% of Texas’s total population. The substantial population growth in urban areas, along with industrial activity, transportation infrastructure, and climate conditions, contributes to high levels of air pollutants^27^. The rural areas of Texas also suffer from poor air quality due to oil and gas production, agricultural activities, and natural dust.

### General approach

**Fig. 2:**
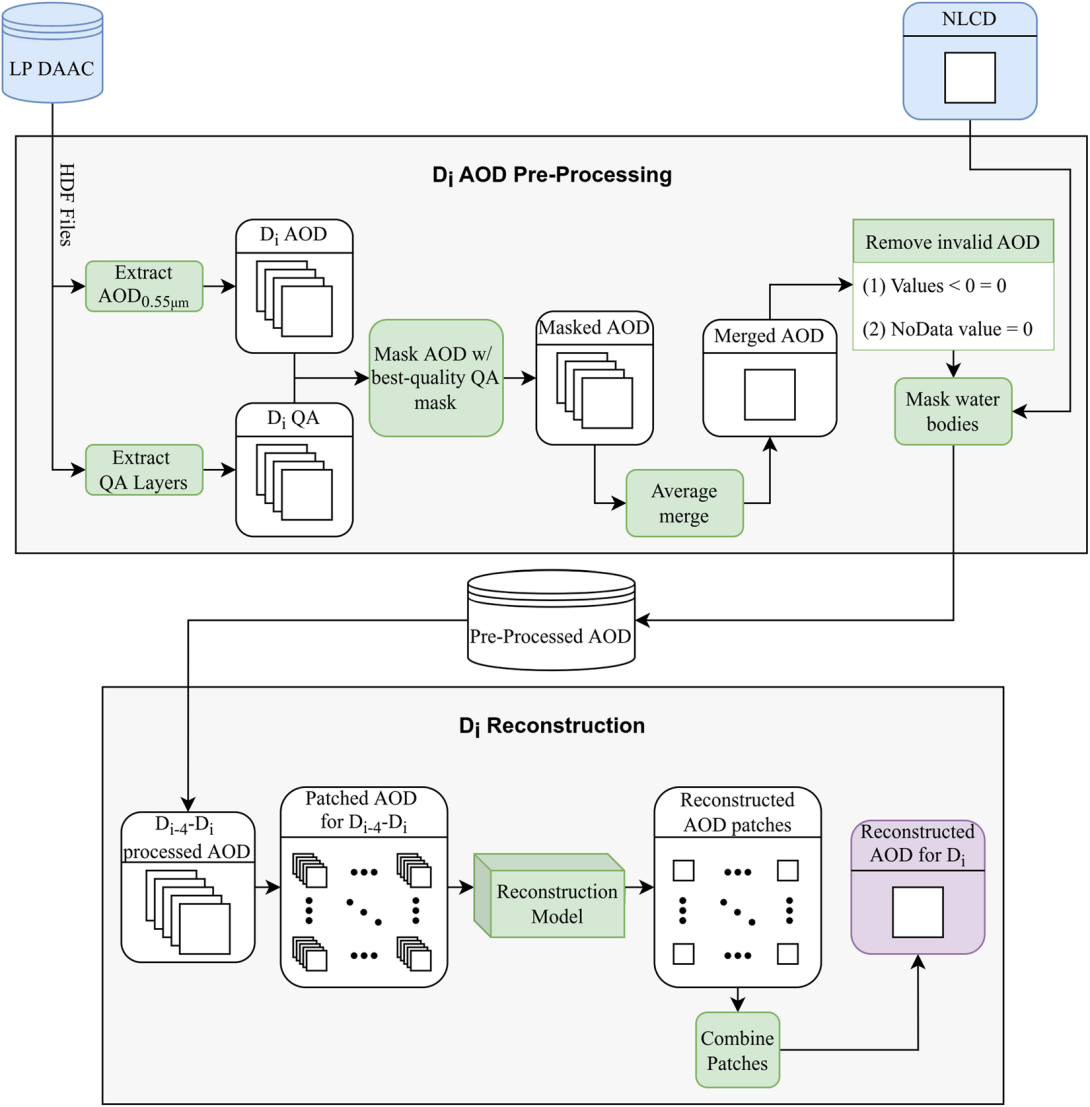
Overview of AOD reconstruction workflow starting from the AOD layer pre-processing to deep learning-based modeling. Green blocks represent functions, blue blocks are inputs, and the single purple block is the reconstructed output. D_i_ denotes the current day in the loop. QA stands for quality assessment.

We begin by presenting an overview of our approach, with subsequent sections providing detailed explanations for each component. The first component involves preprocessing AOD records using low-quality data masking for deep learning modeling (“MAIAC AOD preprocessing” section), followed by the preparation of training and test datasets using realistic simulation (“Training and test dataset creation” section) (Fig. 2). The second component focuses on the model architecture for reconstructing missing or contaminated AOD pixels (“AOD construction” section) (Fig. 2). Lastly, we utilize the reconstructed data to analyze the spatiotemporal patterns of AODs in Texas (“Visualization” section).

#### MAIAC AOD preprocessing

The most recent version of the MCD19A2 daily Land Aerosol Optical Depth (AOD) gridded Level 2 product Version 6 is created from Moderate Resolution Imaging Spectroradiometer (MODIS) Terra and Aqua combined data using the Multi-angle Implementation of Atmospheric Correction (MAIAC) algorithm at a 1 km pixel resolution^28^. Different from its predecessor Dark Target and Deep Blue algorithms, MAIAC uses the semi-empirical Ross-thick/Li-sparse bidirectional reflectance distribution function and the semi-analytical Green’s function solution^28^. To ensure global applicability and operational efficiency, significant improvements have been implemented in various crucial steps such as cloud and snow screening and aerosol-type selection^29^. MAIAC products have gained wide recognition and usage in estimating both natural and anthropogenic aerosol air pollution in various regions worldwide^30–32^.

We obtained all MODIS AOD tiles encompassing the entire state of Texas (H08V05, H08V06, H09V05, H09V06, and H10V05) from January 2010 through August 2022 as a collection of HDF-EOS files from the Land Processes Distributed Active Archive Center (LP DAAC) (https://lpdaac.usgs.gov/news/release-of-modis-version-6-maiac-data-products). We extracted the 550 nm AOD and Quality Assessment (QA) layers from the MODIS AOD tiles. We utilized the 550 nm AOD instead of the 470 nm AOD, as it is a commonly used band for various applications and models^28^. The QA mask consists of the cloud mask and the adjacency mask, which provides information on the cloud level of the current pixel and adjacent pixels. Using the QA layer, we masked the AOD using the best quality flag where both the cloud mask and adjacency mask are clear. This process replaced all pixel values corresponding to a low-quality retrieval with the missing data value. After the cloud masking, we merged the multiple retrievals for each day resulting from the multiple orbit overpasses of the Terra and Aqua satellites. As the retrievals have varying spatial availability, we averaged the retrievals with valid data for each pixel location to obtain a single-band merged output^17^. Typically, there are three to five AOD retrievals available for a single pixel within a day. However, the sample size may be reduced after masking and averaging may introduce basis in representing the daily average. It is important for users to acknowledge this limitation, as detailed in the Usage Notes. We also replaced negative values and the invalid retrieval value with zero, a common practice in deep learning for maximizing the utilization of other useful information^21^. Since retrievals over water are often missing or unreliable^28^, we set those water-covered pixels to zero. The water mask was created from the 2019 National Land Cover Database, which provides major land cover information for the U.S. at a 30-m resolution^33^. This Landsat-based, automated classification mapping product achieved a high overall accuracy of 90.6%. The water class has a particularly high accuracy, with 98% user accuracy and 96% producer accuracy^34^.

After preprocessing, we created a time series of AOD data for subsequent deep-learning modeling. The AOD layer to be reconstructed is defined as D_i_. To form the set of input images, we concatenated the data from the previous four days (D_i-4_) with the current day’s image (D_i_) to create the five-day time series D_i-4_- D_i_. The five-day span was determined experimentally to achieve optimized performance.

#### Training and test dataset creation

To generate sufficient and accurate training samples for the DL-based AOD retrieval, we created artificial masks from real cloud contaminations on MODIS images, which were used to intentionally corrupt the cloud-free AOD layer for realistic simulation. We chose MODIS tile H09V05 which covers large portions of Texas, New Mexico, Colorado, and Utah, and smaller areas of Nevada, Arizona, Kansas, and Oklahoma within the United States for creating these samples (Fig. 1). Each MODIS H09V05 tile was partitioned into 625 image patches of size 48 × 48 pixels (approximately 48 km × 48 km) to limit computational expense during reconstruction. For each day, some image patches were completely cloud-free, while others were cloud-contaminated. We selected all the cloud-free image patches from the years 2020 to 2021 and used them as ground truth in the supervised learning process. In contrast, the corrupted imagery provides realistic examples of the spatial patterns that atmospheric interference may cause. To create the artificial cloud mask for the cloud-free images, we recorded the ratio of corrupted to non-corrupted pixels for each corrupted image and extracted the cloud masks as a binary layer with value one representing corrupted pixels and value zero representing non-corrupted pixels. Using the cloud masks, we artificially corrupted the cloud-free image patches by setting the pixel values corresponding to interference to zero, resulting in each cloud-free retrieval having a corresponding corrupted counterpart. As the model needs a sequential set of multi-day retrievals, these artificially corrupted AOD layers were stacked with their previous four-days retrievals (D_i-4_-D_i_) to form the training dataset. Each training sample was accompanied by a ground truth layer, which was the corresponding cloud-free AOD layer for D_i_. Finally, we generated a total of 78,029 image patches, which were further split into 80% for training (62,423) and 20% for testing (15,606).

### Spatiotemporal LSTM convolutional autoencoder model for AOD reconstruction

#### Model architecture

We developed a novel framework to reconstruct missing AOD satellite retrievals, which addresses the challenge of missing spatially neighboring valid data in many large data gap areas and the dynamic nature of aerosols. To achieve this, we incorporated both spatial and temporal autocorrelations of AOD into the model architecture.

**Fig. 3:**
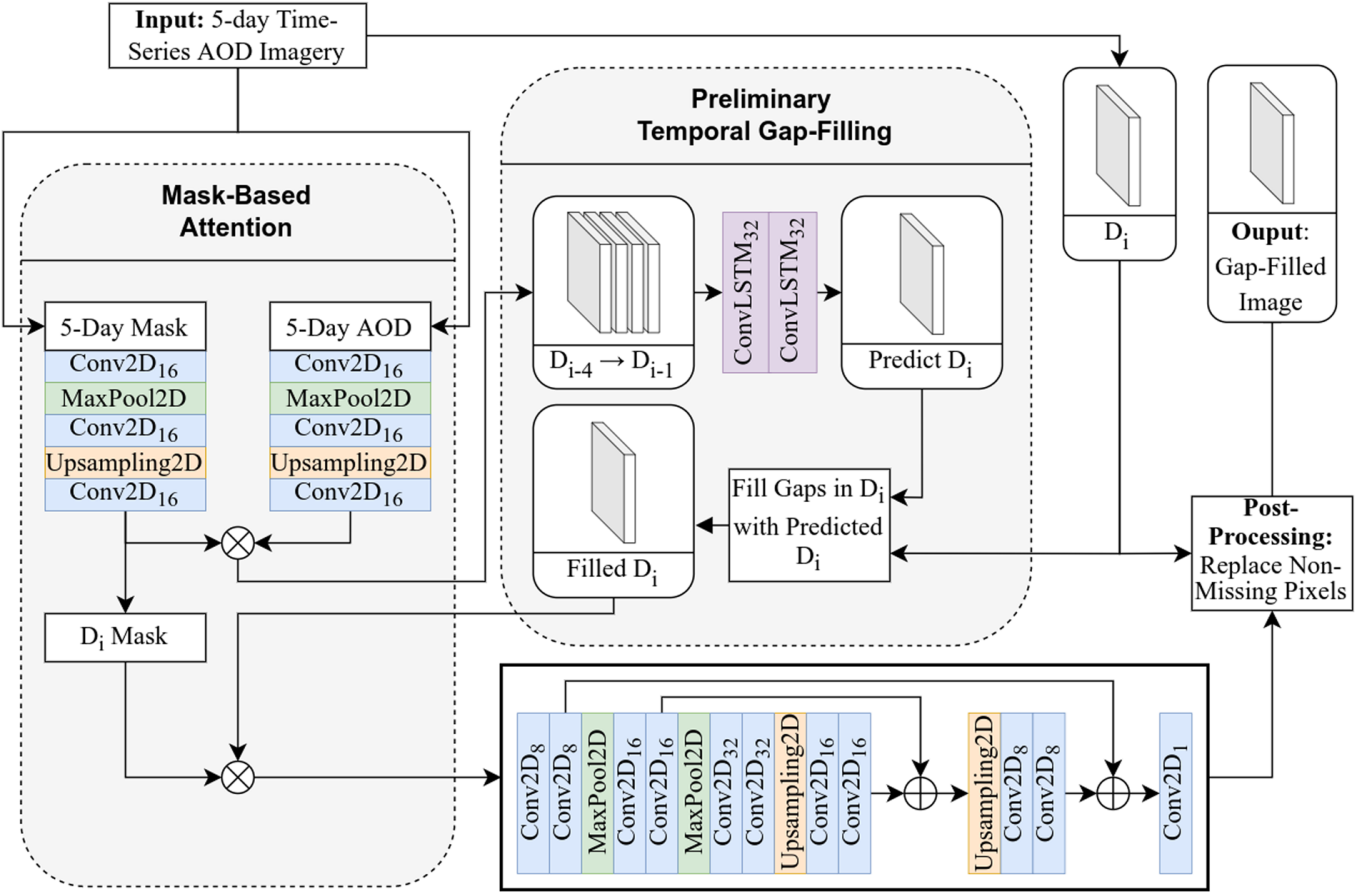
Reconstruction model architecture. D_i_ is the satellite image on the day *i*. Subscripts for convolutional and convolutional LSTM layers denote the number of filters.

The framework begins with a mask-based attention module that includes two small autoencoders: one for the AOD data and another for the missing data mask. The module produces an attention-enhanced representation of the AOD by multiplying the autoencoder outputs (Fig. 3). The goal of this module is to enable the model to learn how to disregard missing pixel values and determine the relative importance of the remaining valid retrieval data for the final prediction.

In the preliminary temporal gap filling, the first four days of attention-enhanced AOD are fed through two convolutional LSTM layers where the input and recurrent transformations are convolutional. Convolutional layers specialize in feature extraction due to their understanding of spatially neighboring pixel patterns and LSTM layers are widely used for sequential predictions due to their ability to “remember” previous inputs for future outputs^35^. When combined, these ConvLSTM layers extract the relevant features from the previous days’ AOD (D_i-4_-D_i-1_) and use these features to predict the AOD values for the target day (D_i_). Using the previous days’ imagery allows the required temporal reconstruction to provide a first approximation of the AOD values at the missing pixel locations and thus minimize the issue of insufficient spatially neighboring valid data. This predicted AOD is then used to fill the missing values in the current day’s retrieval to create a preliminarily complete image. However, as this initial stage has no knowledge of the current day’s valid values, it will be a relatively low-quality prediction due to the dynamic nature of AOD between days.

This initially filled image is then multiplied by the attention mask once again to enhance the focus on the known pixel values which is used as the input to the recurrent convolutional autoencoder. The convolutional layers extract important features to create a more representative image in a multidimensional latent space, while the max pooling layers downsample the representation to reduce the impact of missing data. The upsampling layers use recurrent information that retains high frequency elements from previous steps to restore localized features and return the image to its original resolution. The final convolutional layer converts the multidimensional representation into a single-band AOD layer. Finally, the known values for the current day in the prediction are replaced with their true values in a post-processing step.

#### Model training

Input samples from the training dataset are passed through the model in batches of 64, and the model predicts values for the missing pixels. These predicted values are compared to the ground truths, and the mean squared error (MSE) is used as the loss function to quantify the difference between the predicted and true values. MSE is chosen as the L2 loss function penalizes large errors more heavily than an L1 loss function due to the squaring of the error, resulting in a narrow spread of prediction errors^36^. The loss is then used to update the model weights, and the process is repeated with subsequent batches until all training samples have been used.

1 $$MSE=\frac{1}{n}{\sum }_{i=1}^{n}{\left(ob{s}_{i}-pre{d}_{i}\right)}^{2}$$

Where obs is the observed AOD values and pred is the predicted AOD values. n is the number of total pixels.

### Visualization of spatiotemporal patterns of AOD in Texas

**Fig. 4:**
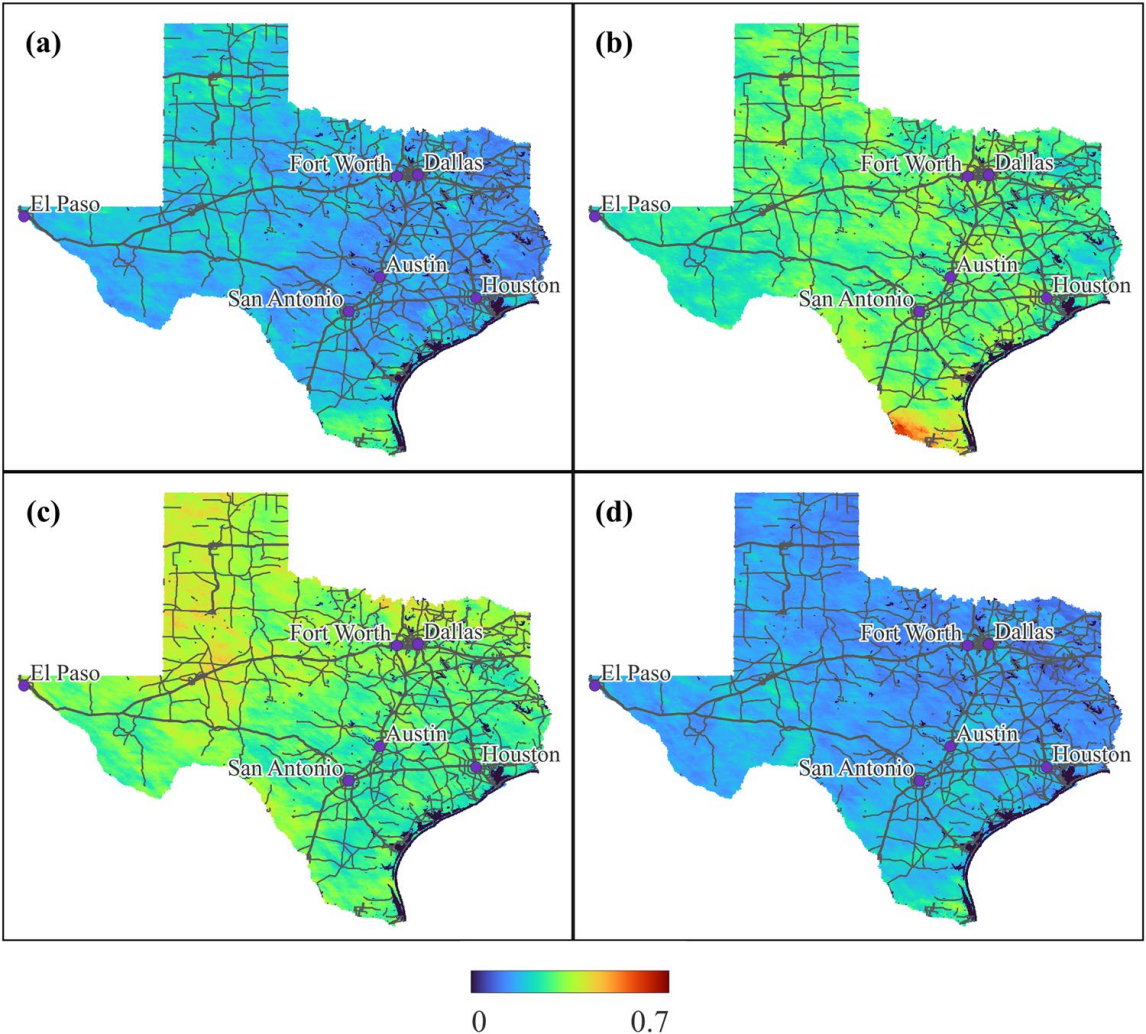
Seasonal patterns of aerosol optical depth across Texas. Visualization of the seasonality of average AOD with each panel a different yearly quarter: (**a**) Jan. - March, (**b**) Apr. - June, (**c**) July - Sept., (**d**) Oct. - Dec. Major cities are labeled and the major roads are represented in lines.

Using the spatiotemporal LSTM convolutional autoencoder model, we have successfully reconstructed daily AOD data covering the entire state of Texas over the past decade. Figure 4 depicts the seasonal AOD patterns observed in Texas throughout the four quarters of the year: January-March, April-June, July-September, and October-December. The AOD can be observed to be relatively high in the northern and western regions confirming the expected high AOD in these areas resulting from the dusty surfaces indicative of these regions. In Fig. 4a,b, an edge can be seen along the southern border of Texas. This artifact is present in the original AOD product, MCD19A2, possibly resulting from the multiple regional aerosol models in this area^28^.

## Data Records

We have made available reconstructed AOD data for Texas at four different time scales: daily, monthly, quarterly, and annual. These datasets have been generated using two data postprocessing approaches to cater to various application needs. The datasets are available at figshare^37–45^.

The daily files are generated using a tiling method, where each tile overlaps the adjacent tiles by a random number of pixels ranging from 6 to 15. Alpha compositing is utilized during merging following reconstruction, creating a smoother gradient at the borders of the tiles. This step is optional but minimizes harsh edges that arise during the tile merging process. The random shift also ensures that composites of multiple days’ data retain a smooth surface gradient, preventing the remaining edges in daily reconstructions from accumulating in the aggregate reconstructed AOD. Data are available at the daily timescale from January 5, 2010, to September 2, 2022. Each daily file is saved as an individual tiff file under the folder “Daily Reconstructed Texas AOD” at figshare^37^.

The aggregated data are presented as monthly, quarterly (quarter-year), and yearly averages. Monthly composites are accessible from January 2010 to August 2022, quarterly composites from the first quarter of 2010 to the second quarter of 2022^38^, and annual composites from 2010 to 2021^39^. Each timescale is available as a zip file and archived under the folders “Monthly Reconstructed Texas AOD”, “Quarterly Reconstructed Texas AOD”, and “Yearly Reconstructed Texas AOD”, separately.

Given that AOD data can be influenced by short-term events like heavy rain or thunderstorms, we generated “smoothly aggregated” data composites to mitigate the impact of temporary disturbances or anomalous measurements, allowing for a more accurate characterization of the average AOD levels. Specifically, within a month, the daily data tails beyond the 2nd and 3rd standard deviation ranges were excluded. This means that data points falling outside the 95% and 99.7% distribution were not considered in the monthly averaging process. Eliminating these extreme values ensures that the resulting data reflect the typical aerosol conditions over the given period. For data processed using the 95% and 99.7% thresholds, the monthly average is saved under the folders of “Monthly Reconstructed Texas AOD (95% threshold)”^40^ and “Monthly Reconstructed Texas AOD (99.7% threshold)”^41^. For the quarterly dataset, we took the average from the two polished monthly datasets and archived them in “Quarterly Reconstructed Texas AOD (95% threshold)”^42^ and “Quarterly Reconstructed Texas AOD (99.7% threshold)”^43^. Yearly datasets averaged from the polished quarterly datasets directly, resulting in data under “Yearly Reconstructed Texas AOD (95% threshold)”^44^ and “Yearly Reconstructed Texas AOD (99.7% threshold)”^45^.

## Technical Validation

### Metric-based validation

We utilized an independent test dataset to demonstrate the model’s capability to accurately predict and generalize to new data. The model performed with an average Root Mean Square Error (RMSE) of 0.017 and an R^2^ value of 0.941 on the test dataset.

2 $$RMSE=\sqrt{\frac{{\sum }_{i=1}^{n}{\left(ob{s}_{i}-pre{d}_{i}\right)}^{2}}{n}}$$

To demonstrate the feasibility and efficiency of the proposed model, we evaluated the resulting images quantitatively at varying levels of corruption using four metrics (Table 1). These metrics included peak signal-to-noise ratio (PSNR), structural similarity index (SSIM), RMSE, and R^2^. PSNR provided ratings based on the entirety of the imagery, while SSIM gave a numerical rating on the perceived similarity of a test image with a reference image, with the assessment based on the structural information of the imagery^46,47^.

3 $$PSNR=20lo{g}_{10}\left(\frac{MA{X}_{AOD}}{\sqrt{MSE}}\right)$$

4 $$SSIM\left(obs,pred\right)=f\left[l\left(obs,pred\right),c\left(obs,pred\right),s\left(obs,pred\right)\right]$$

Where *MAX*_*AOD*_ is the maximum AOD value from the ground truth AOD imagery. *l,c,s* are the luminance, contrast, and structure comparison functions, respectively. The range of *l,c,s* extends from zero to one and the increased value indicates higher similarities for each comparison function. The formulas are defined by:

5 $$l\left(obs,pred\right)=\frac{2{\mu }_{obs}{\mu }_{pred}+{C}_{1}}{{\mu }_{obs}^{2}+{\mu }_{pred}^{2}+{C}_{1}}$$

6 $$c\left(obs,pred\right)=\frac{2{\sigma }_{obs}{\sigma }_{pred}+{C}_{2}}{{\sigma }_{obs}^{2}+{\sigma }_{pred}^{2}+{C}_{2}}$$

7 $$s\left(obs,pred\right)=\frac{{\sigma }_{obs,pred}+{C}_{3}}{{\sigma }_{obs}{\sigma }_{pred}+{C}_{3}}$$

Where *μ*_*obs*_ and *σ*_*obs*_ denote the mean and standard deviation of the true AOD image; *μ*_*pred*_ and *σ*_*pred*_ denote the mean and standard deviation of the predicted AOD image; *C*_1_, *C*_2_, *C*_3_ are constants used to avoid instability when the denominators are close to zero.

**Table 1:** Performance of the reconstruction method using varying metrics in ascending order of corruption level.

Corruption Level	PSNR	SSIM	RMSE	R^2^
**0.01**–**0.1**	67.22	1.000	0.002	0.999
**0.11**–**0.2**	59.45	1.000	0.005	0.997
**0.21**–**0.3**	57.36	0.999	0.007	0.994
**0.31**–**0.4**	55.47	0.999	0.008	0.989
**0.41**–**0.5**	54.00	0.999	0.010	0.987
**0.51**–**0.6**	51.79	0.992	0.013	0.980
**0.61**–**0.7**	50.12	0.992	0.016	0.972
**0.71**–**0.8**	48.53	0.992	0.019	0.957
**0.81**–**0.9**	43.98	0.992	0.032	0.905
**0.91**–**1.0**	39.57	0.907	0.053	0.555

All metrics remained relatively stable at levels indicating good performance from low to median levels of corruption. However, a drop in accuracy became evident at higher levels of corruption, ranging from 50% to 80%. At the highest level of corruption (80%–99%), the model’s performance degraded significantly in terms of image similarity, although the RMSE remained relatively low at 0.053.

### Visual assessment

**Fig. 5:**
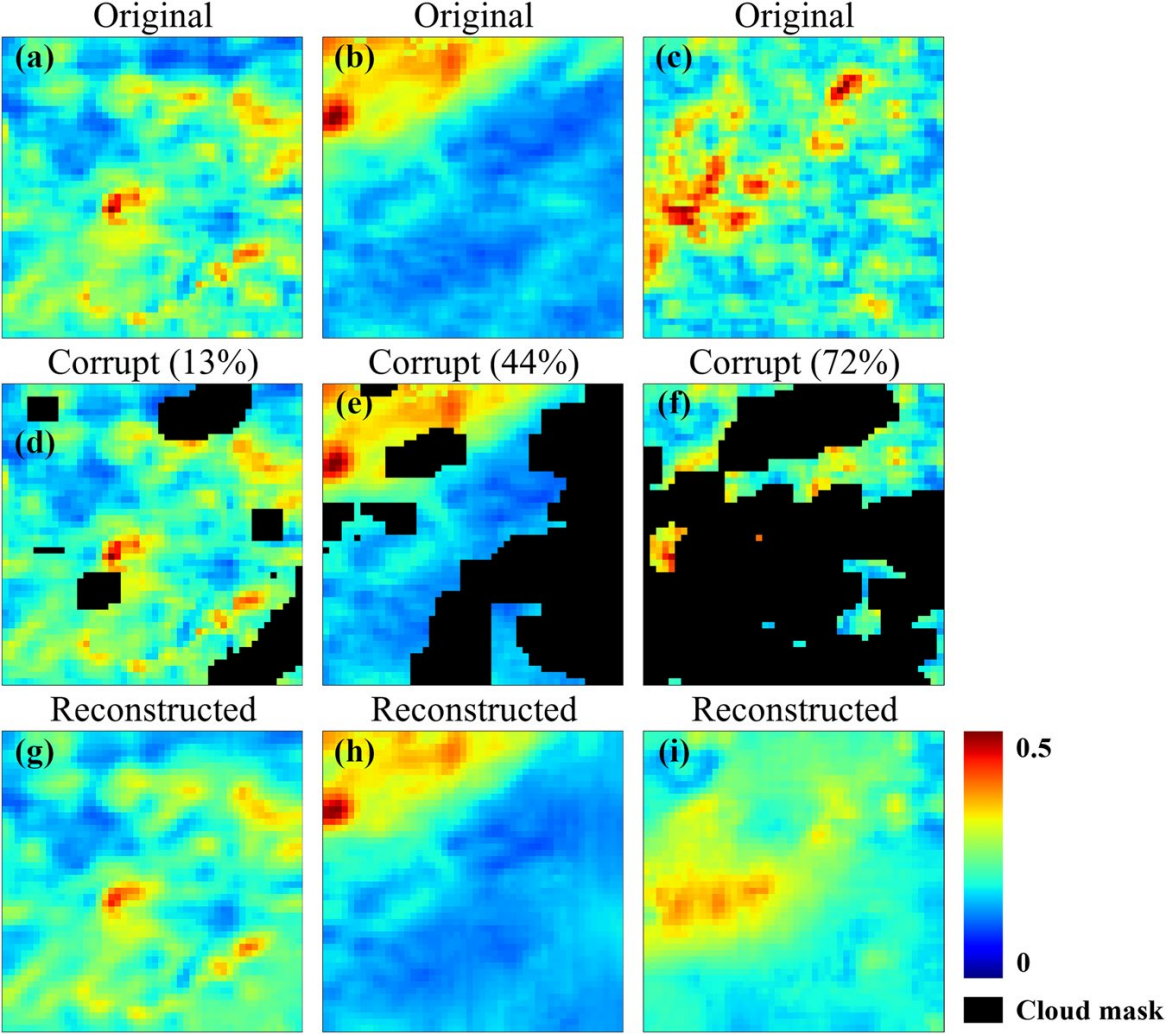
Reconstructed AOD at varying cloud covers before post-processing. Each image covers an area of approximately 48 km × 48 km. The corruption percentages are the ratio of corrupted pixels to the total number of pixels. (**a**–**c**) are the original images from three sample locations, (**d**–**f**) are the corresponding corrupted counterparts (i.e. the current day for the model input), and (**g**–**i**) are the model’s reconstructed output.

We provided visual examples of the reconstruction performance in Fig. 5, which consists of three trios with varying percentages of missing data. The first row displays the original cloud-free images (Fig. 5a–c), followed by their corrupted counterparts, which served as the current day’s input to the model (Fig. 5d–f). The final row shows the model’s reconstructed output before post-processing (Fig. 5g–i). Artificially corrupted images were used for illustration purposes instead of true-corrupted images, to enable visual comparison with the ground truth. The reconstructed images showed high similarity to the original images, even at the relatively high corruption level of 72%. While some high-frequency localized AOD information loss was expected, most of the major features were retained.

### Pixel-wise comparison

**Fig. 6:**
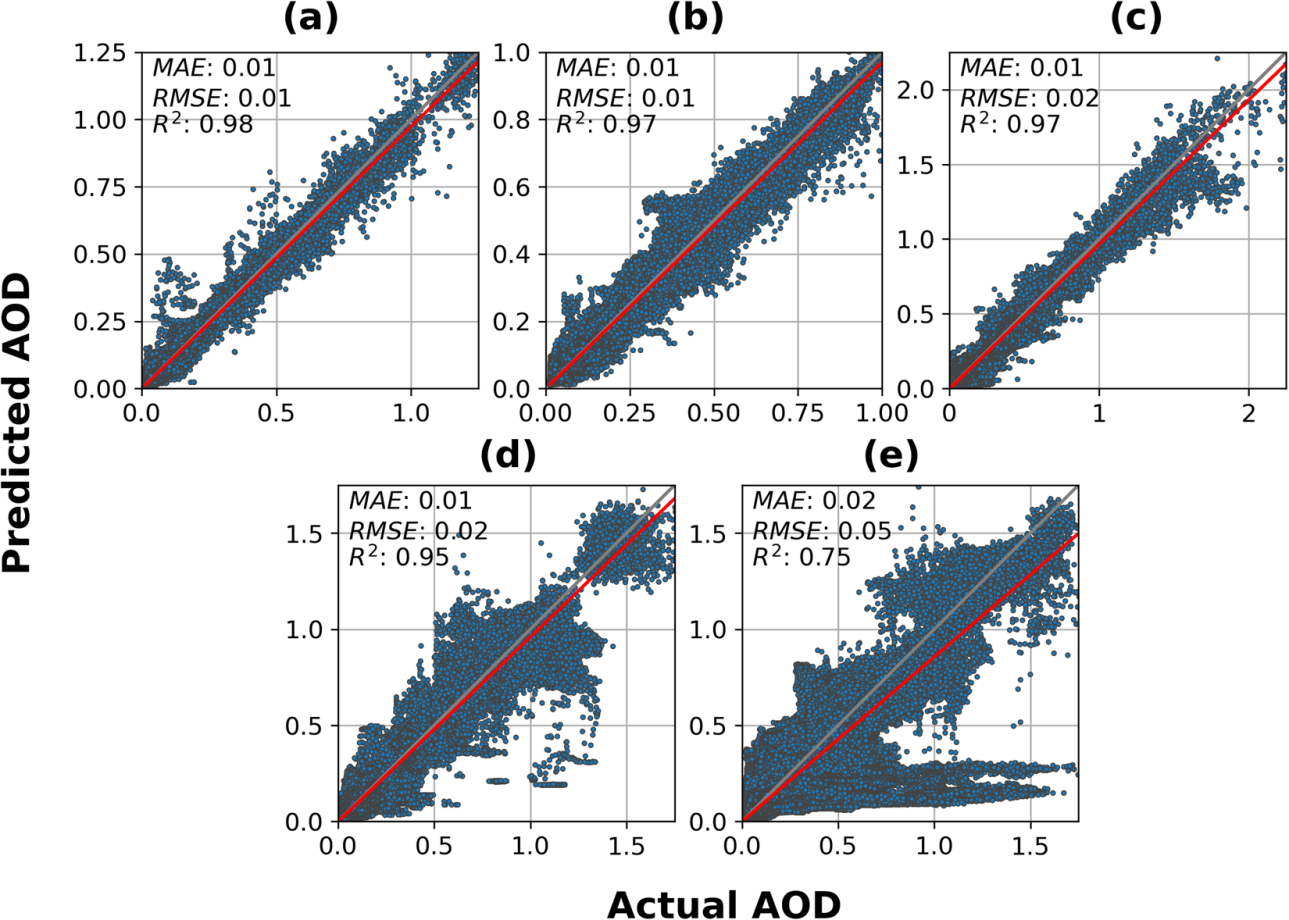
Scatter plot showing test dataset actual vs. predicted AOD for the reconstruction model. The one-to-one line is shown as a grey line, and the regression line is shown as a red line. The subplots each correspond to a different corruption level: (**a**) 1%–20%; (**b**) 21%–40%; (**c**) 41%–60%; (**d**) 61%–80%; (**e**) 81%–100%.

We conducted a pixel-wise comparison of the predicted AOD with the reference AOD across the spectrum of reference AOD concentrations at varying corruption levels (Fig. 6). Each point in the scatter plot represents a pixel-wise comparison between the predicted image and the target image. Three metrics, namely mean absolute error (MAE), RMSE, and R^2^, were calculated to quantify the degree of correlation. The spread of errors, measured by the tightness around the one-to-one line, remained relatively high for 1% to 60% corruption levels (Fig. 6a–c), indicating consistently low deviations in the predictions from the truth. For 61% to 80% corruption levels, the spread of errors became wider but still maintained a strong trend with the one-to-one line (Fig. 6d). At the highest levels of corruption (81–100%) containing very little valid data (Fig. 6e), the error spread remained similar to the previous corruption level except for some pixels whose predictions remained around a constant value between 0.1 and 0.4 at all AOD levels. These pixels were likely present in images with the highest corruption levels (near 100% corruption). In these cases, there was no spatial data available for predictions, and little to no information was present in the previous days’ imagery for temporal reconstruction, as occurs during long periods of thick, persistent cloud coverage. The trend lines for each corruption level also showed that the model tended to underpredict slightly at 1–80% corruption levels (Fig. 6a–d) and more significantly at the highest levels of corruption.

### Validation against AERONET

**Fig. 7:**
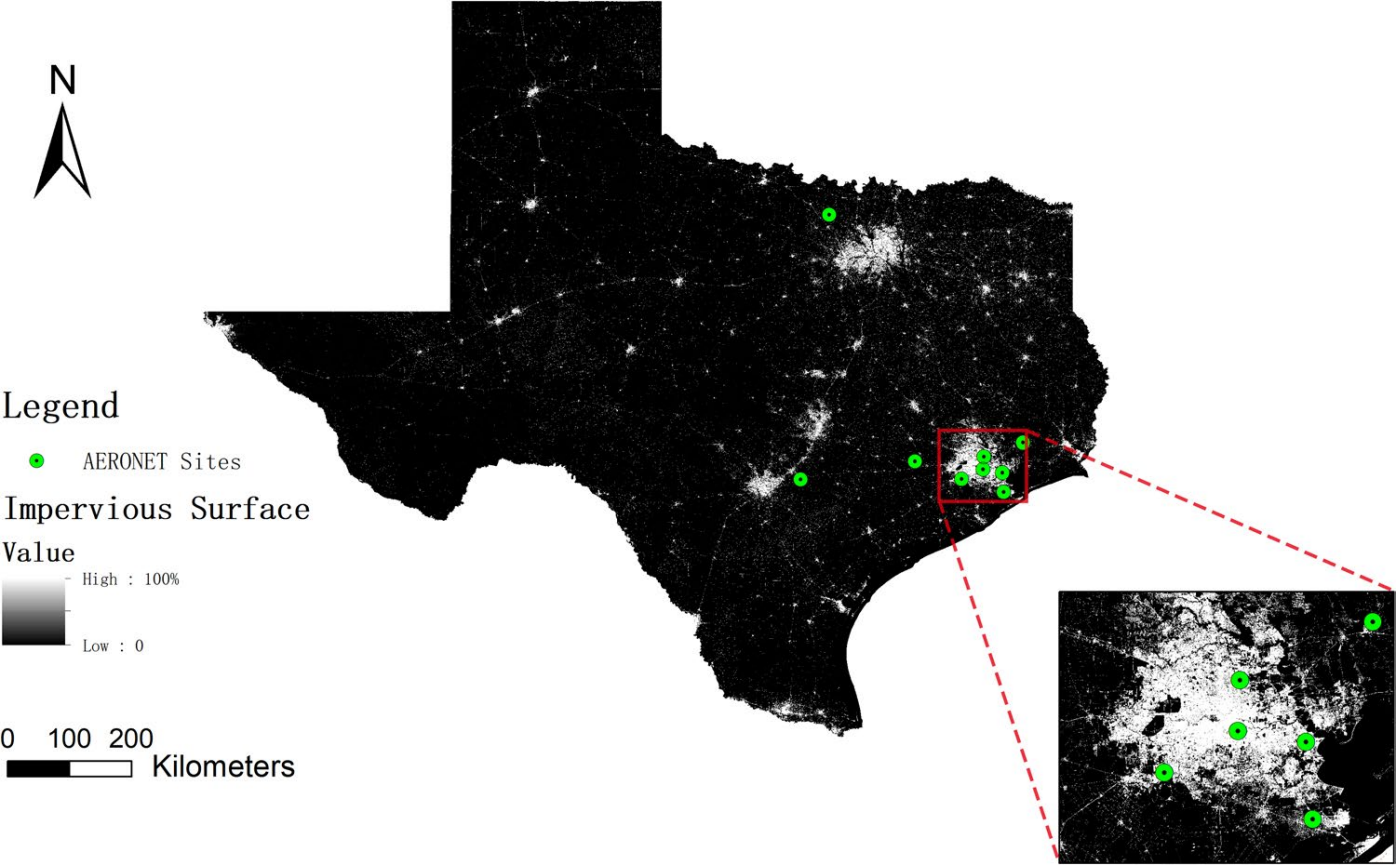
Distribution of AERONET sites in Texas.

We utilized the latest AERONET Version 3.0 Level 2.0 AOD dataset to validate our AOD data product using all Texas sites (Fig. 7). To ensure consistency, we interpolated the AERONET data to a wavelength of 550 nm (*AOD*_550_) using the closest available wavelengths of 440 nm and 675 nm^48^:

8 $$AO{D}_{550}=AO{D}_{500}{\left[\frac{550}{500}\right]}^{-A{E}_{440-675}}$$

Where AE represents the Angström Exponent, indicating aerosol size distribution^48,49^. Due to the coarser resolution of satellite-derived AOD data, we used square buffers with 1 × 1 km², 3 × 3 km², 9 × 9 km², 18 × 18 km², and 55 × 55 km², as well as a circular buffer with a radius of 27.5 km around each AERONET site, to determine the spatial scale at which satellite data can best represent station data accurately. These buffer zones were selected because they have been used in previous research^50–53^. Accuracy metrics, including the linear regression intercept and slope, Pearson’s correlation coefficient (R), RMSE, and the bias were used. We also used three Expected Error (EE) envelopes, representing an accuracy of ±1σ (i.e. 68% of the retrievals), ±2σ, and ±3σ, to provide a visual and numerical representation of the acceptable range of errors. Data points falling within the envelopes are within the expected error range.

9 $${\rm{EE}}5=\pm \left(0.05+0.05\cdot {\rm{AOD}}\_{\rm{AERONET}}\right)$$

10 $${\rm{EE}}10=\pm \left(0.05+0.10\cdot {\rm{AOD}}\_{\rm{AERONET}}\right)$$

11 $${\rm{EE}}15=\pm \left(0.05+0.15\cdot {\rm{AOD}}\_{\rm{AERONET}}\right)$$

12 $${\rm{AOD}}\_{\rm{AERONET}}-\left|{\rm{EE}}\right|\le {\rm{AOD}}\_{\rm{MAIAC}}\le {\rm{AOD}}\_{\rm{AERONET}}+\left|{\rm{EE}}\right|$$

**Fig. 8:**
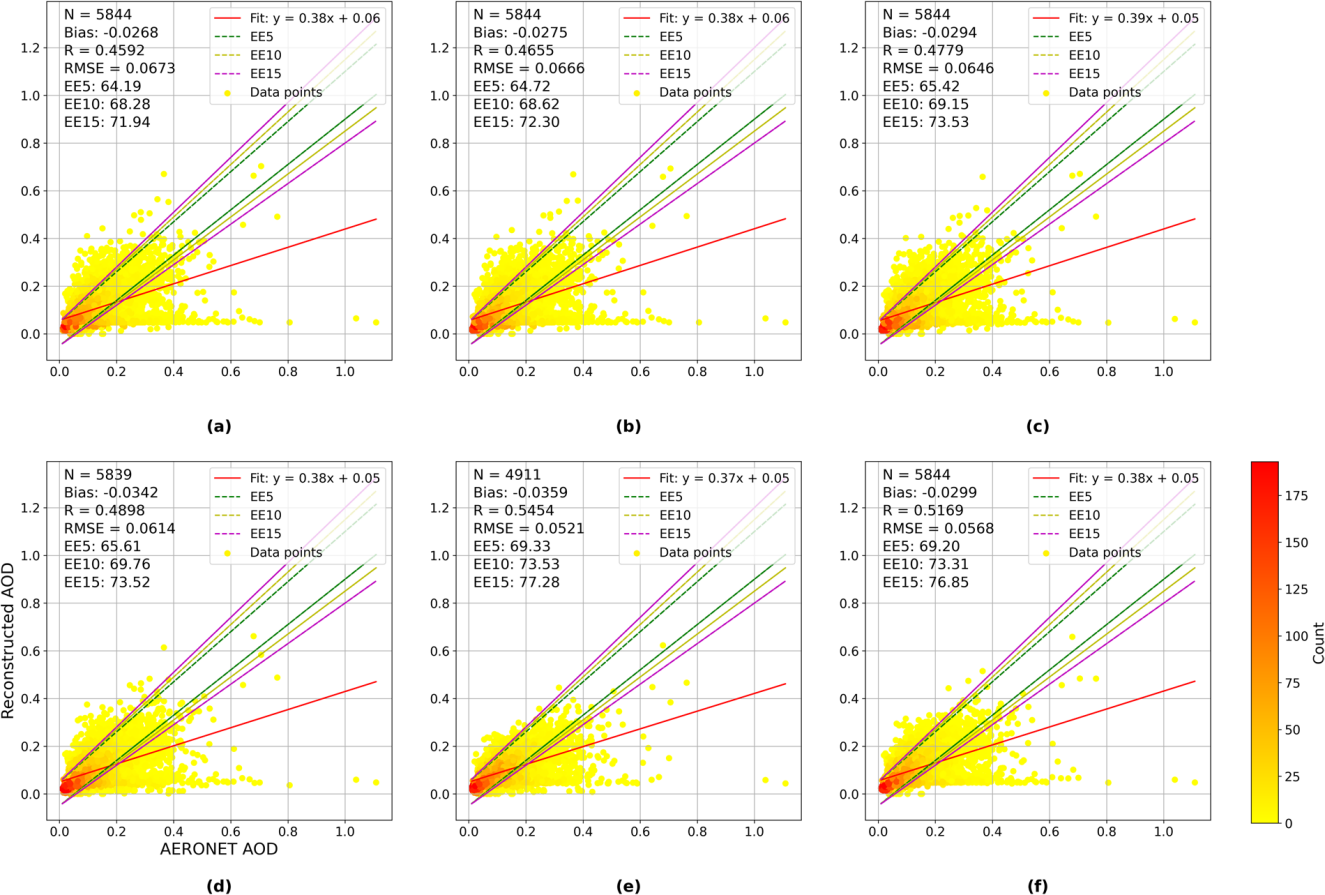
Comparison between collocated AERONET and reconstructed MAIAC AOD at varying buffer sizes: (**a**) 1 × 1 km², (**b**) 3 × 3 km², (**c**) 9 × 9 km², (**d**) 18 × 18 km², (**e**) 55 × 55 km², (**f**) 27.5 km radius circle. N: the total count of MAIAC-AERONET data pairs. EE: expected error.

Figure 8 illustrates the correlation between AERONET AOD across all Texas sites with data during our study period and the reconstructed AOD retrieval over the six different spatial buffers, where R values range from 0.46 to 0.55. It is important to note that these sites are relatively clustered and situated near the Greater Houston area (Fig. 7). MAIAC has been documented to exhibit low accuracy over bright surfaces^28^ and the overall performance of MAIAC AOD is expected to be higher. In addition, the minimal bias ranging from −0.027 to −0.036 and the RMSE values ranging from 0.052 to 0.067 suggest systematic and controlled error. The data within the EE5, EE10, and EE15 error envelopes provide additional validation of the product’s reliability. The EE15 achieved an inclusion rate of 77.28% at the 55 km buffer, underscoring the accuracy and adaptability of our reconstructed AOD data at larger spatial scales.

## Usage Notes

While MODIS measurements are acquired at specific times of the day, prior research has demonstrated that MODIS data can effectively represent daily average optical thickness regardless of particle size or the optical thickness range^54^. MODIS’s insensitivity to the time of day is likely attributed to the relatively long aerosol lifetime, spanning several days, which minimizes differences between morning and afternoon measurements. Furthermore, aerosol loading is heavily influenced by the frequency of synoptic scale meteorological processes, which are largely unaffected by diurnal cycles.

It is important to note, however, that the cloud screened data from MODIS tend to exhibit a bias toward less cloudy and drier conditions^54^, as observed in regions with such climate conditions, such as Texas. For studies conducted in more humid regions, researchers should exercise greater caution when using the MODIS data to investigate diurnal cycles. Furthermore, areas located near diurnal source regions (e.g., urban centers), should also consider the suitability of the data for their specific purposes.

## Data Availability

All code for processing the raw MCD19A2 HDF-EOS files as well as reconstructing the missing data is available on GitHub: https://github.com/lu-liang-geo/AOD-reconstruction or Figshare^55^. The code is all provided in Python using open-source libraries for reproducibility.
